# *Rickettsia slovaca* “spotted fever”

**DOI:** 10.1016/j.jdcr.2023.06.032

**Published:** 2023-06-29

**Authors:** Pascal Del Giudice, Mathieu Reverte, Christian Boissy, Pierre Edouard Fournier

**Affiliations:** a Infectiology and Dermatology Unit, Centre Hospitalier Intercommunal de Fréjus-Saint-Raphaël, Fréjus, France; b Laboratoire d’anatomopathologie Médipath, Fréjus, France; c Laboratoire des rickettsies, Institut Infectio-Méditerrannée, Centre Hospitalier Universitaire Marseille, Hôpital la Timone, Marseille, France

**Keywords:** *Rickettsia*, rickettsiosis, *Ricketssia slovaca*

## Introduction

*Rickettsia slovaca* is a human rickettsiosis that has been associated with a specific clinical syndrome, scalp eschar and neck lymph adenopathy after tick bite (SENLAT).^1^^,^^2^ Patients with this disease typically present with a single scalp eschar following a tick bite associated with a cervical lymphadenopathy. To our knowledge, the first proven case of human infection with *R. slovaca* was reported in 1997 in France.^3^ Subsequently, Raoult et al^1^ described the SENLAT syndrome as a “spotless Rickettsiosis caused by *R. slovaca* and associated with dermacentor ticks.” We observed one patient who had a *R. slovaca* infection presenting as a spotted eruption. Consent for the publication of patient photographs and medical information was provided by the authors at the time of article submission to the journal stating that the implicated patient gave consent for their photographs and medical information to be published in print and online and with the understanding that this information may be publicly available.

## Case report

**Fig 1:**
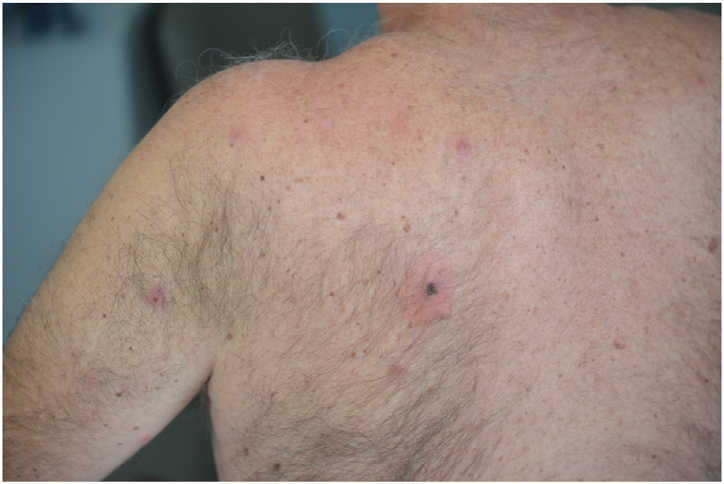
An overview of the eschar and some erythematous papules.

**Fig 2:**
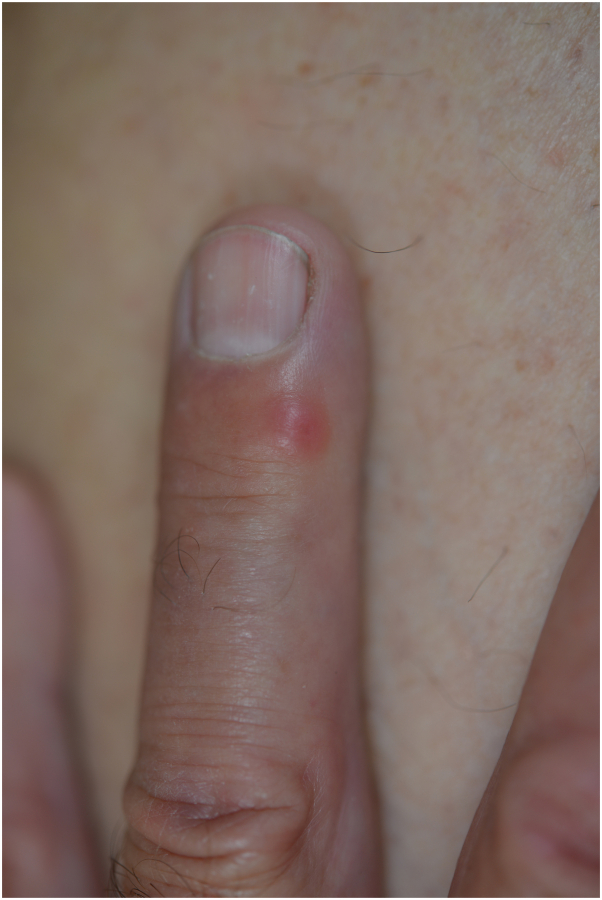
Close-up view of erythematous papule on finger.

**Fig 3:**
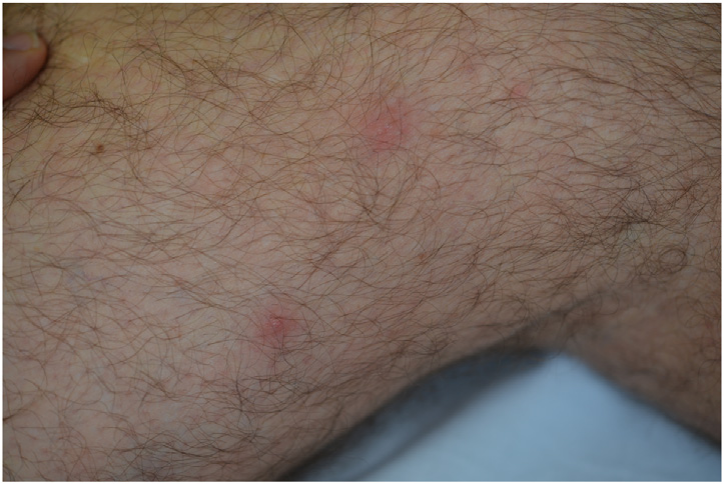
Close-up view of erythematous papules on the thigh

A 63-year-old man, residing in the Var county (city of Lorgues), southern France, presented with a disseminated papular skin eruption in April 2022. He had removed a tick from his back 5 days earlier. His clinical examination showed a dorsal eschar at the tick bite site surrounded by an inflammatory zone as well as about 20 painless erythematous papules disseminated all over the body, involving the back (Fig 1), limbs (Fig 2), and thigh (Fig 3). The patient had no fever, and his general condition was not altered. A diagnosis of rickettsiosis was suspected, and the patient was treated with doxycycline at a dosage of 200 mg per day for 1 week.

**Fig 4:**
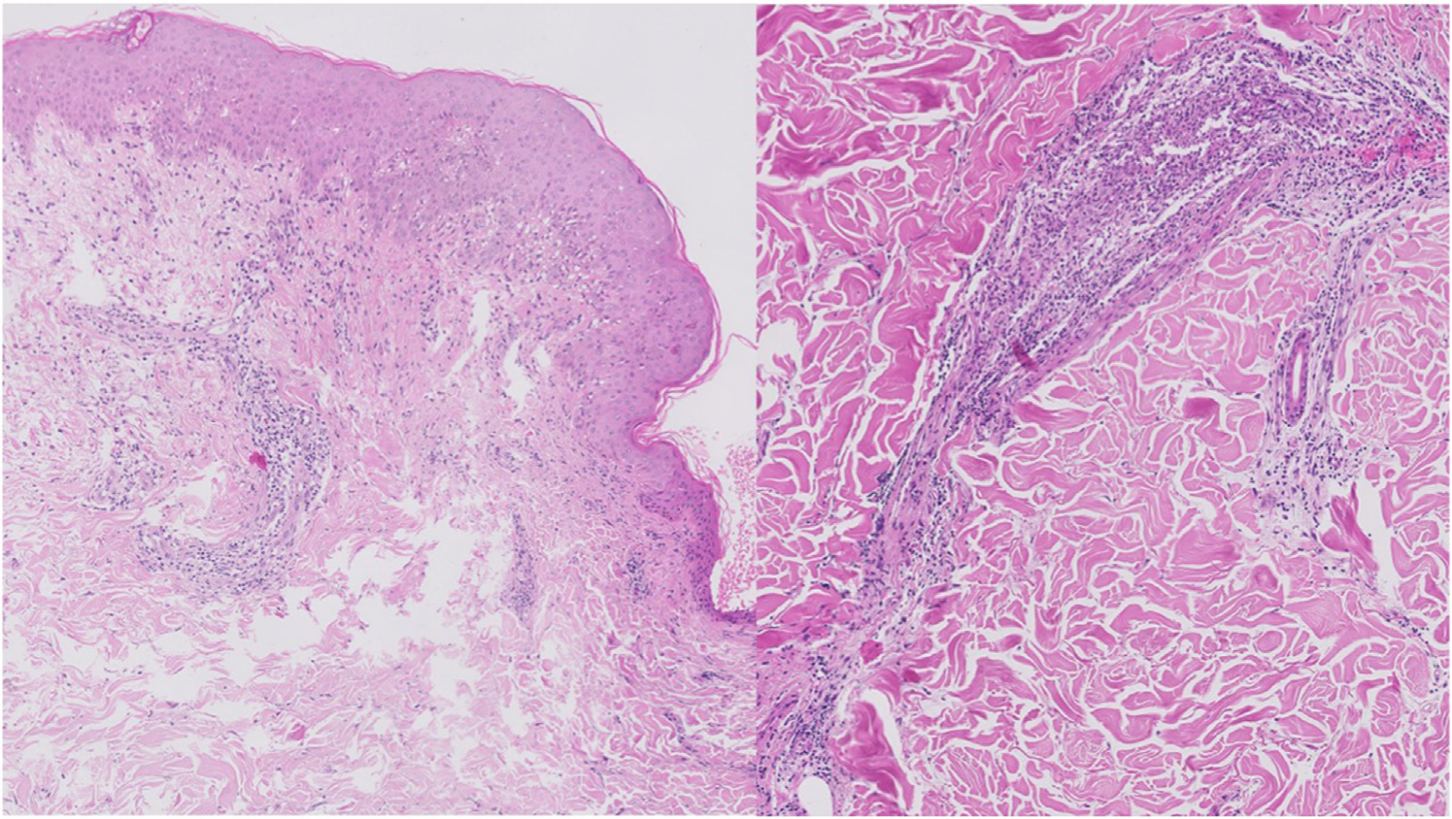
Biopsy of a papule: Hematoxylin-eosin stain (original magnification: ×100): polymorphic inflammatory cells in dermis with moderate exocytosis in epidermis.

The skin biopsy of the eschar showed an epidermal ulceration with a fibrin-rich inflammatory exudate. Under the ulceration, there was an edema with ectasic capillaries and an inflammatory infiltrate with polymorphic cells (neutrophils, lymphocytes, histiocytes, and rare eosinophils) in the dermis and hypodermis with moderate exocytosis in the epidermis. Skin biopsy of a papule (Fig 4) showed slight epidermal hyperplasia with orthokeratosis. There was an edema of the papillary dermis and a mostly perivascular infiltrate of lymphocytes and histiocytes (rare neutrophils), with moderate exocytosis in the epidermis and no necrotic features.

DNA extracted from an eschar smear using an EZ1 automate (Qiagen, Hilden Germany) was positive for *Rickettsia slovaca* based on the results of real-time polymerase chain reaction (PCR), whereas the first serology performed the same day was negative for *Rickettsia slovaca*. The evolution was favorable with disappearance of the lesions in a few days.

## Discussion

Raoult et al^1^, ^2^, ^3^ described the SENLAT syndrome as a “spotless rickettsiosis” caused by *R. slovaca*. The disease is associated with cervical lymphadenopathy and sometimes low-grade fever. It can heal spontaneously. No serious clinical form or complication has been reported,^1^, ^2^, ^3^ and such a rickettsial disease could be treated with the administration of doxycycline or macrolides antibiotics for a few days. If performed too early, in the acute phase of infection, the serology could be negative, as in our case. In the acute phase of the disease, the sampling of the eschar for PCR may confirm the diagnosis by showing the presence of *R. slovaca*.

Subsequently, SENLAT has been reported to be associated with other *Rickettsia* species and bacteria.^4^

In the following years, 2 types of other cutaneous manifestations have been described exceptionally: facial cellulitis or edema and skin eruption. Indeed, Parola et al^4^ reported facial edema associated with SENLAT in 2009.

Although initially described as a “spotless rickettsiosis,” few cases of skin eruptions have been reported in *R. slovaca* infections.^1^ Cazorla et al^5^ observed a case of “papular rash consisting of 10 pink spots on the thorax and arms.” Méchaï et al^6^ described a patient with *R. slovaca* SENLAT presenting “maculopapular rash all over the body.” Kostopoulou et al^7^ described a patient without “any sign of a tick bite such as an eschar or lymphadenopathy; however, 2 days after admission, he presented a maculopapular rash on the face, thorax, and extremities, including the palms and soles.” Sayfullin et al^8^ reported a “vesicular-papular rash on his limbs and trunk concentrated around the eschar and on the skin of the knee.” Unfortunately, in all these cases, no detailed clinical description was provided, no pictures of the eruption nor histology of the papules were available. To our knowledge, only Tappe et al^9^ observed a 62-year-old woman who had a typical SENLAT, followed by maculopapular exanthema, 39°C fever, knee arthralgias, and back pain 2 days later. The author provided images of a slight macule isolated from the leg and scattered erythematous papules on the forehead.^9^ The prevalence of such spotted eruptions in *R. slovaca* infection appears to be extremely rare. Indeed, Silva-Pinto et al^10^ have reviewed 537 cases of *R. slovaca* infection and found that 13 (0.2%) had a rash.

To our knowledge, we provide the first detailed clinical and histological description of the “spotted” eruption in a patient infected with *R. slovaca.* Further reports are welcome to clearly delineate the dermatological spectrum of this infection.

## Conflicts of interest

None disclosed.
